# Amb a 1 isoforms: Unequal siblings with distinct immunological features

**DOI:** 10.1111/all.13196

**Published:** 2017-06-14

**Authors:** M. Wolf, T. E. Twaroch, S. Huber, M. Reithofer, M. Steiner, L. Aglas, M. Hauser, I. Aloisi, C. Asam, H. Hofer, M. A. Parigiani, C. Ebner, B. Bohle, P. Briza, A. Neubauer, F. Stolz, B. Jahn‐Schmid, M. Wallner, F. Ferreira

**Affiliations:** ^1^ Department of Molecular Biology University of Salzburg Salzburg Austria; ^2^ Biomay AG Vienna Competence Center Vienna Austria; ^3^ Department of Pathophysiology and Allergy Research Medical University of Vienna Vienna Austria; ^4^ Laboratory for Immunological and Molecular Cancer Research Paracelsus Medical University Salzburg Austria; ^5^ Department of Biological, Geological, and Environmental Sciences University of Bologna Bologna Italy; ^6^ Allergy Clinic Reumannplatz Vienna Austria

**Keywords:** allergenicity, Amb a 1 allergen, cross‐reactivity, immunogenicity, ragweed pollen allergy

## Abstract

**Background:**

Ragweed pollen represents a major allergy risk factor. Ragweed extracts contain five different isoforms of the major allergen Amb a 1. However, the immunological characteristics of Amb a 1 isoforms are not fully investigated. Here, we compared the physicochemical and immunological properties of three most important Amb a 1 isoforms.

**Methods:**

After purification, the isoforms were physicochemically characterized, tested for antibody binding and induction of human T‐cell proliferative responses. Their immunological properties were further evaluated in vitro and in vivo in a mouse model.

**Results:**

Amb a 1 isoforms exhibited distinct patterns of IgE binding and immunogenicity. Compared to Amb a 1.02 or 03 isoforms, Amb a 1.01 showed higher IgE‐binding activity. Isoforms 01 and 03 were the most potent stimulators of patients’ T cells. In a mouse model of immunization, Amb a 1.01 induced higher levels of IgG and IgE antibodies when compared to isoforms 02 and 03. Interestingly, ragweed‐sensitized patients also displayed an IgG response to Amb a 1 isoforms. However, unlike therapy‐induced antibodies, sensitization‐induced IgG did not show IgE‐blocking activity.

**Conclusion:**

The present study showed that naturally occurring isoforms of Amb a 1 possess different immunogenic and sensitizing properties. These findings should be considered when selecting sequences for molecule‐based diagnosis and therapy for ragweed allergy. Due to its high IgE‐binding activity, isoform Amb a 1.01 should be included in diagnostic tests. In contrast, due to their limited B‐ and T‐cell cross‐reactivity patterns, a combination of different isoforms might be a more attractive strategy for ragweed immunotherapy.

**Abbreviations**
ACN: acetonitrile
AIT: allergen‐specific immunotherapy
ALUM: aluminum hydroxide
Amb a: *Ambrosia artemisiifolia*
AP: alkaline phosphatase
BSA: bovine serum albumin
CD: circular dichroism
CD23: cluster of differentiation 23
CPM: counts per minute
DPM: disintegrations per minute
DTT: dithiothreitol
EBV: Epstein‐Barr virus
FA: formic acid
FAB: facilitated antigen binding
FITC: fluorescein isothiocyanate
FTIR: Fourier transform infrared
HPLC: high‐performance liquid chromatography
IL: interleukin
IFN: interferon
MS: mass spectrometry
MS/MS: tandem mass spectrometry
NaP: sodium phosphate buffer
PBMC: peripheral blood mononuclear cell
PBS: phosphate‐buffered saline
PE: phycoerythrin
RBL: rat basophilic leukemia cells
TCC: T‐cell clones
TBS: Tris‐buffered saline
TFA: trifluoroacetic acid
SDS‐PAGE: sodium dodecyl sulfate–polyacrylamide gel electrophoresis

## 1 INTRODUCTION

Worldwide, pollen of short ragweed is recognized as a major allergy risk for atopic individuals. Epidemiological studies showed that 23% to 32.8% of the US population is sensitized to ragweed, whereas prevalence of sensitization in European countries varies between 3.5% (eg*,* Italy) and 54% (eg, Hungary).^1^, ^2^, ^3^ A study in northern China showed that 6.5% of allergic patients responded positive to ragweed pollen in skin prick tests.^4^ In South America, 23% of Colombian patients with acute asthma displayed ragweed‐specific IgE antibodies.^5^

More than 95% of ragweed pollen‐allergic patients display IgE antibodies against the major allergen Amb a 1, which is a member of the pectate lyase family.^6^ Allergenic pectate lyases have also been identified as major allergens in the pollen of *Cupressaceae* trees such as Mediterranean cypress, mountain cedar, as well as Japanese cedar and cypress. Interestingly, IgE cross‐reactivity between ragweed and *Cupressaceae* pollen‐derived pectate lyases has been reported to be relatively low.^7^ To date, five different Amb a 1 isoforms have been acknowledged by the WHO/IUIS allergen nomenclature subcommittee (www.allergen.org) showing sequence homologies between 63% and 87% (Fig. S1B).^8^

Previous studies showed that isoforms of certain major allergens display distinct immunological properties. For example, isoforms of the major house dust mite allergen Der p 2 diverge only by 3% in their amino acid sequences, but were shown to differ in their IgE‐binding properties and to induce different cytokine patterns upon stimulation of PBMCs from allergic and nonallergic donors.^9^ Similarly, two isoforms of Bet v 1 with sequence identity of 96% showed striking differences in their IgE‐binding properties and in their capacity to activate T cells from allergic patients.^10^ These differences seem to be linked to the capacity of Bet v 1 isoform 0102 to form cysteine‐linked aggregates^11^ and its fold dynamics. These properties, which were demonstrated to be critical for binding to cathepsin S and for efficient processing,^12^ are not shared by the Bet v 1.0101 isoform.

Recent reports suggested that Amb a 1 isoforms might display distinct antibody binding properties.^6^, ^8^ This prompted us to analyze in great detail the allergenic as well as immunogenic properties of Amb a 1 isoforms. Understanding the immunological and allergenic properties of individual isoforms comprising natural Amb a 1 is of major importance for the development of adequate and efficient products for diagnosis and therapy of ragweed pollen allergy.

## 2 MATERIALS AND METHODS

### 2.1 Protein purification

Natural Amb a 1 was purified from 6 g of *Ambrosia artemisiifolia* pollen (Batch: 020511204 purchased from Allergon AB, Ängelholm, Sweden). Recombinant Amb a 1.03 was produced in the yeast *Pichia pastoris* and purified from culture supernatants. Methods are described in detail in Appendix S1.

### 2.2 Peptide analysis by nano‐LC‐MS/MS

Analyses of tryptic peptides obtained from ragweed pollen extracts and from purified isoforms were performed as described in Appendix S1.

### 2.3 Physicochemical characterization

Physicochemical analyses were performed as described in Appendix S1.

### 2.4 Patients′ sera

Ragweed‐allergic patients included in the study were identified based on typical case history, positive skin prick test, and ImmunoCAP analysis (CAP w1 common ragweed *Ambrosia artemisiifolia,* Phadia, AB, Uppsala, Sweden) (Table S1). Experiments using anonymized serum samples from allergic patients were approved by the ethics committee of the Medical University of Vienna (Nr. 712/2010).

#### 2.4.1 Proliferation assays: PBMC and TCC

T‐cell recognition of Amb a 1 isoforms was assessed using peripheral blood mononuclear cells (PBMCs) and Amb a 1‐specific T‐cell clones (TCC) isolated from ragweed‐allergic patients.^13^ A detailed description is provided in Appendix S1. Experiments using anonymized blood samples from allergic patients were approved by the ethics committee of the Medical University of Vienna (Nr. 497/2005).

### 2.5 Immunoblots and ELISA

Immunoblots and ELISA experiments were performed according to the established protocols. A detailed description is provided in Appendix S1.

### 2.6 Mediator release assays

Mediator release assays were performed using RBL‐2H3 cells transfected with the alpha chain of the human IgE receptor^14^ and passively sensitized with IgE antibodies from ragweed‐allergic donors as described in Appendix S1.

### 2.7 Facilitated antigen‐binding assay (FAB)

To determine Amb a 1 isoform‐specific blocking capacity of patients’ IgG and IgG of immunized mice toward Amb a 1‐specific human IgE, the FAB assay^15^, ^16^ was used. A detailed description is given in Appendix S1.

### 2.8 Animal experiments

Female BALB/c mice (Janvier Labs, Saint‐Berthevin, France) at the age of 8 weeks were used for experiments (five animals/group). 5 μg of antigen was either adsorbed to 50% Alu‐Gel‐S (Serva, Heidelberg, Germany) v/v or formulated without adjuvant in PBS pH 7.4, and administered as two 50 μL s.c. injections, bilaterally in the lumbar region at days 0, 7, 14, and 21. Sera were taken at days ‐2, 14, and 28. Specific IgG1 and IgG2a antibodies were analyzed by ELISA as described in Appendix S1. IgE antibodies were analyzed in mediator release assays. All animal experiments were conducted according to National guidelines approved by the Austrian Ministry of Science (BMWF‐66.012/0009‐II/3b/2013).

## 3 RESULTS

### 3.1 Characterization of natural Amb a 1

Mass spectrometric analyses of ragweed pollen extracts showed that Amb a 1 comprised 78% of the allergen content (data not shown). Similar results were reported in other studies.^6^, ^17^ In addition, our analyses revealed that the natural Amb a 1 mixture consisted of 33% of isoform 01, 16% of isoform 02, 11% of isoform 03, 6% of isoform 04, and 34% of isoform 05 (Table S2). As isoforms 01, 02, and 03 were shown to display higher IgE‐binding activity than isoforms 04 and 05,^8^ they were selected for further characterization.

### 3.2 Purification and physicochemical characterization of Amb a 1 isoforms

**Figure 1:**
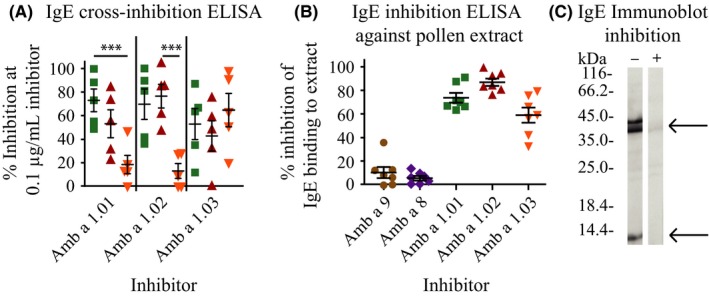
IgE binding to Amb a 1 isoforms analyzed by cross‐inhibition ELISA to coated Amb a 1 isoforms (A) and ragweed extract (B). IgE immunoblot of ragweed extract using a serum pool from 10 allergic patients with (+) or without (−) preincubation with a mixture of equal amounts of Amb a 1 isoforms 01, 02, and 03 (C). The arrows indicate full‐length natural Amb a 1 and the high IgE‐binding beta chain, respectively. The beta chain is produced by proteolysis of Amb a 1 during pollen extraction

Isoforms 01, 02, and 03 were purified from ragweed pollen extracts by standard chromatography. Isoform 03 was also produced as a recombinant protein in *P. pastoris*. The purified Amb a 1 isoforms were able to inhibit between 59% and 87% of the IgE binding to ragweed pollen extracts, as determined in inhibition ELISA and immunoblot experiments (Figure 1B,C). After purification, the identity, purity, and isoform composition of the preparations were confirmed by mass spectrometry (Fig. S2). The nAmb a 1.01 preparation was found to consist of 94% isoform 01, 4% isoform 04, and 1% isoform 02. The nAmb a 1.02 preparation consisted of 86% isoform 02 and 12% isoform 01. The natural preparation of Amb a 1.03 contained 87% of isoform 03 and 8% of isoform 01. Recombinant isoform 03 had a purity of >99%. Using sera from ragweed‐allergic patients in immunoblots, cross‐inhibition ELISA, and mediator release assays, no significant differences in the IgE‐binding properties of recombinant and natural isoform 03 could be detected (Fig. S3). Due to low purification yields of the natural form (40 μg/g pollen), the recombinant form of isoform 03 was selected for further experiments. As determined by the HEK‐Blue™ mTLR4 reporter cell assay, the levels of endotoxin in isoforms 01 and 03 preparations were below the detection limit of 6.1 EU/mg, whereas for Amb a 1.02 a value of 166 EU/mg was measured.

The secondary structure elements of the three Amb a 1 isoforms were analyzed using CD and FTIR (Fig. S4). The CD spectra showed the typical shape of proteins with high beta‐sheet content in combination with a large portion of unordered structures. The CD patterns indicated that each isoform possessed distinct structural features, which was further confirmed by FTIR experiments. The major structural element in the family 1 of polysaccharide lyases (www.cazy.org) is a central parallel beta‐helix. This is clearly indicated by the observed high beta‐sheet content (35.6%‐40.5%) in all isoforms. The molecules also displayed variable content of alpha helical structures, that is, 11.5%, 22.7%, and 15.4% for isoforms 01, 02, and 03, respectively. Based on these results, it seems reasonable to suggest that the overall structures of Amb a 1 isoforms differ significantly (Fig. S1C).

### 3.3 Amb a 1.01 shows higher IgE‐binding activity than isoforms 02 and 03

**Figure 2:**
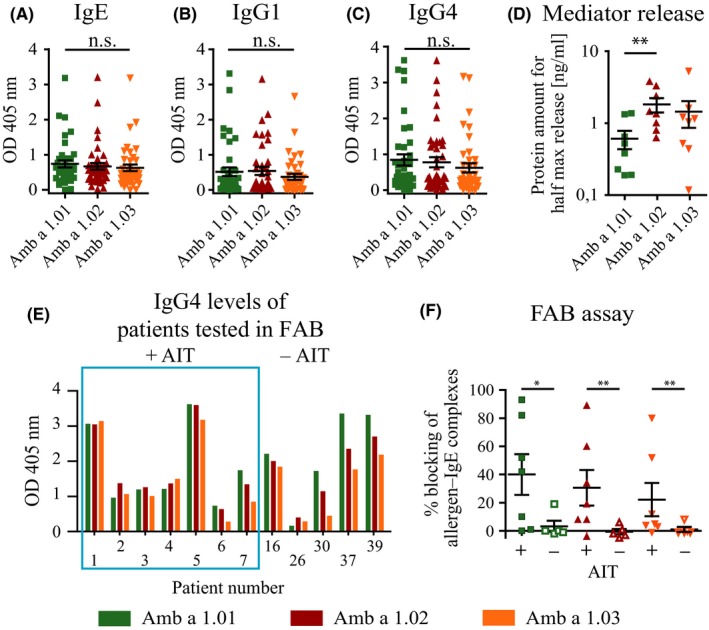
Patients′ (n=39) antibody binding to Amb a 1 isoforms was analyzed by ELISA (A‐C) and mediator release assays (D) (n=8 patients). Amb a 1‐specific IgG4 levels in patients’ sera included in FAB assays (E). Facilitated antigen‐binding (FAB) assays (F). +AIT, ragweed‐allergic patients subjected to allergen‐specific immunotherapy; −AIT, nontreated ragweed‐allergic patients. Statistics were calculated by ANOVA,* P*<.05 was considered significant

Antibody‐binding profiles of 39 ragweed‐allergic patients were analyzed by immunoblot and ELISA assays. Of the 39 patients, seven individuals received ragweed‐specific immunotherapy (+AIT). In immunoblots, the majority (87%) of the patients reacted with Amb a 1 (Fig. S5A). ELISA experiments showed that 97% of the patients were sensitized to Amb a 1 (Fig. S5B), but no significant differences in the binding of IgE, IgG1, or IgG4 to the tested Amb a 1 isoforms could be detected (Figure 2A‐C). However, in mediator release assays, isoform 01 was found to react significantly stronger than the other isoforms. 0.61, 1.83, and 1.46 ng/mL of isoforms 01, 02, and 03, respectively, were necessary to induce half‐maximal release (Figure 2D).

### 3.4 Amb a 1‐specific IgG4 antibodies from non‐AIT patients do not show IgE‐blocking activity

The IgE‐blocking activity of sensitization‐ versus therapy‐induced IgG antibodies was evaluated using the FAB assay. We selected five non‐AIT patients and compared them with seven AIT patients. All patients (non‐AIT and AIT) showed high levels of Amb a 1‐specific IgG4 recognizing each isoform. In six of seven AIT patients, IgG‐blocking activity could be detected, but their blocking capacity did not correlate with IgG antibody levels. Remarkably, in contrast to therapy‐induced IgGs, sensitization‐induced specific IgGs showed no IgE‐blocking activity (Figure 2E,F).

### 3.5 Amb a 1.01 shows high immunogenicity in mice

**Figure 3:**
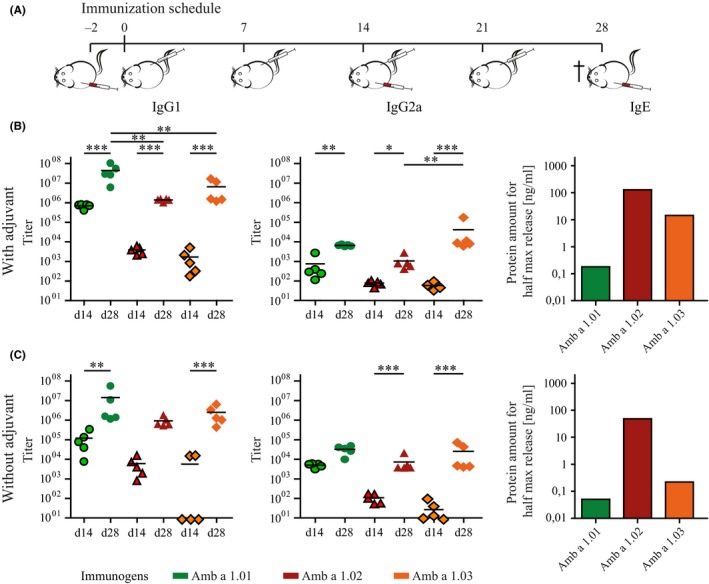
(A) Scheme of animal immunizations. Mice (n=5/group) were immunized either with antigen adsorbed to ALUM as adjuvant (B) or with antigen alone (C). IgG1 and IgG2a titers were determined by ELISA. Functional IgE was determined in mediator release assays using serum pools (day 28) of each group. Statistical analyses were performed with ANOVA;* P*<.05 was considered significant

To investigate the immunological properties of Amb a 1 isoforms in vivo, mice were immunized using two immunization schedules, with and without ALUM as adjuvant (Figure 3A). In both immunization protocols, isoform 01 was the most potent antigen inducing the highest IgG1 titers. Moreover, the onset of the immune response against isoform 01 was faster compared to the other tested antigens. In the presence of ALUM, immunization with isoform 03 induced higher IgG2a titers than with isoforms 01 or 02, whereas in the absence of ALUM, higher titers were observed with isoform 01 (Figure 3B,C; Table S3).

### 3.6 Amb a 1.01 isoform is a very potent allergen

In mediator release assays, we found that adsorption to ALUM had only minor effects on the induction of IgE specific for isoforms 01 and 02, whereas the IgE response against isoform 03 was boosted in the absence of ALUM. Both in the presence and in the absence of ALUM, isoform 01 was the most potent inducer of IgE, as indicated by the low protein amounts (0.05 ng protein/mL without ALUM; 0.18 ng protein/mL with ALUM) needed to trigger half‐maximal mediator release in basophils (Figure 3B,C).

### 3.7 Isoform‐induced antibodies show limited cross‐reactivity

The degree of cross‐inhibition between the individual isoforms was investigated using sera from ragweed‐allergic patients. Isoform 01 showed the highest cross‐IgE inhibition capacity followed by isoform 02. Isoform 03 showed very low capacity of cross‐inhibition (Figures 1A, Fig. S6).

**Figure 4:**
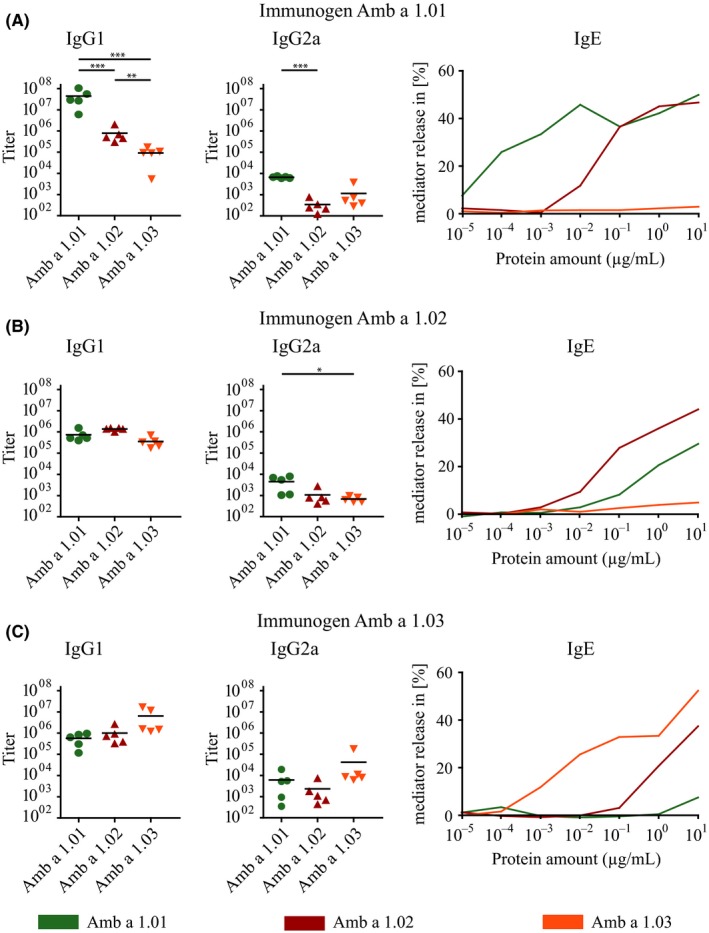
Cross‐reactivity of IgG1 and IgG2a antibodies in mice immunized with Amb a 1.01 (A), Amb a 1.02 (B), or Amb a 1.03 (C) was analyzed by ELISA using sera collected at day 28. Serum titers of individual mice are presented as scatter plots. Statistical analyses were performed with ANOVA. *P*<.05 was considered significant. Mouse IgE cross‐reactivity was analyzed by mediator release assays using RBL cells passively sensitized with sera from immunized mice

Cross‐reactivity was further investigated in the mouse immunization model. Isoform 01‐induced antibodies (IgG1 and IgG2a) showed very little cross‐reactivity with the other two isoforms (Figure 4A). In contrast, immunization with isoform 02 induced highly cross‐reactive antibodies (Figure 4B). Antibodies induced by isoform 03 were moderately cross‐reactive (Figure 4C). Similar to the results obtained in human IgE cross‐inhibition ELISA, sera from single isoform‐immunized mice showed limited cross‐reactivity when stimulated with other isoforms in RBL mediator release assays (Figure 4). Taken together, the results indicate that both IgE and IgG antibodies induced by natural sensitization with Amb a 1 isoforms in humans or by immunization in mouse display distinct patterns of epitope recognition.

### 3.8 Proliferation assays with PBMC

**Figure 5:**
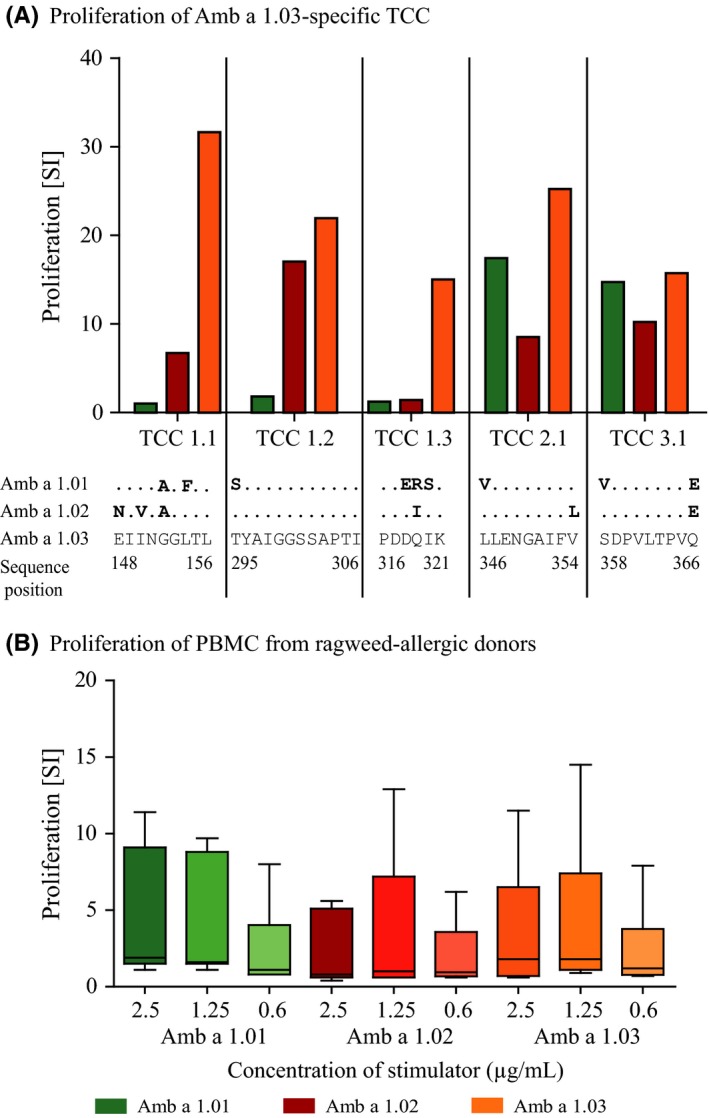
Proliferation responses to Amb a 1 isoforms. Five Amb 1.03‐specific TCC isolated from three ragweed pollen‐allergic patients (patient 1: TCC1.1, TCC1.2, TCC1.3; patient 2: TCC2.1; patient 3: TCC3.1) (A) and PBMCs from seven ragweed pollen‐allergic donors (B) were stimulated with Amb a 1 isoforms. Epitope specificities are shown below the respective TCC (A). SI values (ratio between the cpm of stimulated cultures and the cpm of unstimulated cultures) are shown. Background proliferation of TCC and PBMC in medium ranged from 1418 to 3743 cpm and from 1555 to 18470 cpm, respectively

Amb a 1 isoforms were tested for their capacity to induce proliferation in PBMCs from seven ragweed‐allergic patients. PBMCs were stimulated with three different concentrations (0.6, 1.25, and 2.5 μg/mL) of Amb a 1 isoforms. Higher and stronger proliferative responses were obtained upon stimulation with Amb a 1.01 and 1.03, when compared with Amb a 1.02 (Figure 5B).

### 3.9 Amb a 1.03‐specific T‐cell clones

Five Amb a 1.03‐reactive TCC obtained from three different ragweed‐allergic donors were specific for five independent epitope regions of Amb a 1.03 (epitope sequences are indicated in Figure 5A).^13^ Four of five TCC also reacted to Amb a 1.02 and only two of five to Amb a 1.01. These results suggest that at the T‐cell level, isoforms 02 and 03 have the highest cross‐reactivity level, in conformity with their higher sequence identity.

### 3.10 Mouse antibodies block human IgE binding to Amb a 1 in an isoform‐specific manner

**Figure 6:**
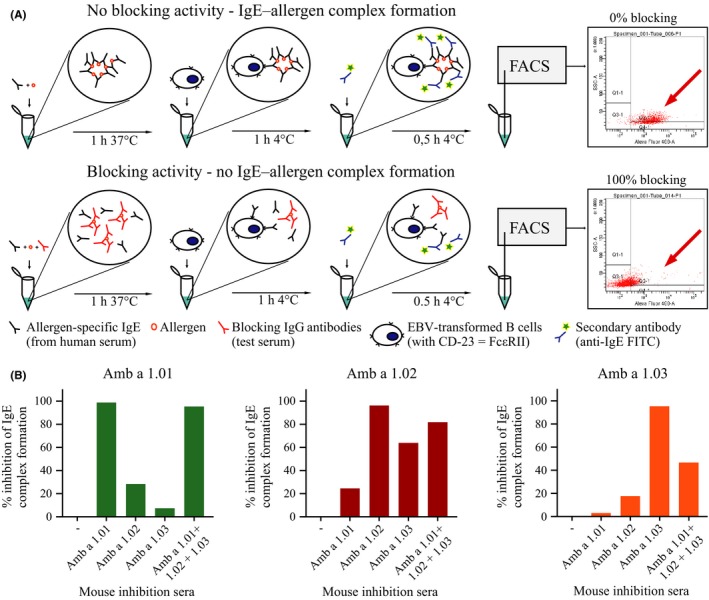
Schematic representation of the FAB assay (A) The inhibitory capacity of mouse immunization‐induced IgG antibodies was tested with sera of Amb a 1.01‐, Amb a 1.02‐, or Amb a 1.03‐immunized mice with the respective Amb a 1 isoforms. A pool of ragweed‐allergic patients′ sera was used as an indicator for the IgE complex formation (B)

To analyze the epitope profile and IgE‐blocking capacity of mouse antibodies induced by immunization with Amb a 1 isoforms, we performed FAB assays (schematic in Figure 6A). Mouse sera were incubated with the different isoforms and a pool of human indicator serum containing high levels of Amb a 1‐specific IgE antibodies. IgE–allergen complex formation was determined by flow cytometry and expressed as % of cells carrying such complexes. For isoform 01, 90.9% cells carried such complexes, 76.3% for isoform 02 and 86% for isoform 03. After the addition of serum from isoform 01‐immunized mice, the percentage of cells carrying IgE–isoform 01 complexes decreased to 1.1%, demonstrating high self‐blocking capacity (98.8%). Similarly, sera from isoforms 02‐ and 03‐immunized animals achieved almost 100% blocking activity against their respective complexes (96.3% and 95.6%, respectively). In contrast, sera from isoforms 02‐ and 03‐immunized mice showed very limited capacity (28.6% and 7.7%, respectively) to block the formation of IgE–isoform 01 complexes. Sera from isoform 01‐immunized mice showed very low blocking activity against isoforms 02 and 03 (24.8% and 3.6%, respectively). Experiments using combinations of isoform‐specific mouse sera showed that only an equal mix of sera containing antibodies against all three isoforms resulted in high blocking capacity (Figure 6B).

## 4 DISCUSSION

Amb a 1, the most abundant allergen in ragweed pollen, is composed of a mixture of five isoforms with amino acid sequence identities ranging between 63% and 87%. The clinical relevance of Amb a 1 has been documented in numerous publications: (i) A study by Zeiss et al. 18 showed that as much as 13% of the total serum IgE in ragweed‐allergic patients is specific for Amb a 1; (ii) the content of Amb a 1 (formerly antigen E) in pollen extracts nicely correlates with the potency of the extract, as determined by skin prick tests in ragweed‐allergic patients^19^; (iii) immunotherapy with purified Amb a 1 was shown to be as effective as whole ragweed extract in reducing symptoms induced by pollen exposure.^20^ Thus, clinical data strongly suggest that despite the fact that during the sensitization process individuals develop IgE antibodies to several molecules found in ragweed extracts, from the point of view of patients’ immunotherapy, Amb a 1 represents the most important allergen. In fact, in contrast to Amb a 1 isoforms, our experiments demonstrated that the minor allergens Amb a 8 and Amb a 9 display very low IgE inhibition capacity toward coated ragweed pollen extract. In these experiments, isoforms 01 and 02 showed the highest capacity to inhibit IgE binding (up to 87% of total ragweed‐specific IgE) to coated ragweed pollen extract. As our results presented here as well as published data showed that Amb a 1 isoforms 01, 02, and 03 bind most of the patients′ ragweed‐specific IgE,^8^, ^21^ our study focused on the characterization of these three isoforms.

Amb a 1‐allergic patients receiving AIT showed high levels of IgG (especially IgG4) against all Amb a 1 isoforms, which is an expected effect of the therapy. However, many of the non‐AIT patients showed Amb a 1‐specific IgG1 and more surprisingly also IgG4 antibodies. Recent studies with large birth cohorts have also found IgG antibodies to a wide variety of allergens.^22^, ^23^ However, in the study by Schwarz et al., IgG4 antibodies against airborne allergens were rarely found.^22^ In this respect, it should be noted that in the MAS birth cohort study, antibodies have been tested at the age of 2 years.^22^ This could provide an explanation for discrepancies on the detection of allergen‐specific IgG4 antibodies, as IgG4 antibody production seems to depend on repeated antigen stimulation for longer periods of time.^24^ Nevertheless, the occurrence of allergen‐specific IgG4 in treated and nontreated ragweed‐allergic patients raises the question on the beneficial effects (eg*,* blocking activity) attributed to this antibody class in immunotherapy.^25^ Our results using functional FAB assay revealed that only AIT‐induced IgG antibodies had a blocking effect on IgE. In contrast, sensitization‐induced IgGs were not able to block specific IgE–Amb a 1 interactions. In line with a recent study by Hoh et al. describing somatic hypermutations and affinity maturation of IgG4 during immunotherapy,^26^ our results argue for an important role of IgG4 in the outcome of immunotherapy and suggest the use of a functional assay capable of measuring the IgE–IgG interplay^27^ to monitor patients undergoing allergen‐specific immunotherapy.

To further investigate the sensitization capacity of the different Amb a 1 isoforms, we immunized mice in the presence and absence of ALUM as adjuvant. Isoform 01 was the most potent sensitizer, inducing very high levels of IgG and IgE antibodies, followed by isoform 03. However, isoform 01‐specific antibodies were less cross‐reactive than antibodies induced by immunizations with isoform 02 or 03. Interestingly, the allergenic properties of Amb a 1 isoforms were not significantly influenced by adsorption to ALUM as adjuvant. The differences in the cross‐reactivity patterns of the isoforms might be due to distinct surface characteristics. Indeed, three‐dimensional models showed that the surface of each isoform has a unique fingerprint of charged and polar patches (Fig. S1C), which in turn could result in distinct antibody‐binding epitopes. In the mouse model, isoform 02 showed the highest level of cross‐reactivity, whereas in cross‐inhibition ELISA experiments with human sera, isoform 01 had the greatest inhibitory capacity toward the other isoforms.

The capacity of the different Amb a 1 isoforms to induce proliferation of T cells from ragweed‐allergic donors was assessed in PBMCs and Amb a 1‐specific TCC. Amb a 1 01 and 03 were the most potent stimulants of PBMCs, which is in good agreement with their higher sensitizing capacity in our mouse immunization models. Interestingly, Amb a 1.03‐specific TCC showed very limited cross‐reactivity upon stimulation with isoform 01. Thus, limited cross‐reactivity between isoforms 01 and 03 is observed both at B‐ and T‐cell level, in humans and in mice. In general, despite its low allergenicity/immunogenicity, isoform 02 showed high degree of B‐ and T‐cell cross‐reactivity.

Taken together, our results raise the question whether a single Amb a 1 isoform would be sufficient for a molecule‐based approach in ragweed immunotherapy. Consequently, successful AIT for ragweed‐allergic patients will likely depend on the careful selection of Amb a 1 isoforms for a therapeutic cocktail. In principle, the variable surfaces of the Amb a 1 isoforms could explain the differences observed in their antibody cross‐reactivity properties and sensitization capacity. However, further experiments are needed to unequivocally demonstrate the relationship between the isoform structures and their immunogenicity/allergenicity.

## CONFLICT OF INTEREST

F. Ferreira is a member of Scientific Advisory Boards (HAL Allergy, NL; AllergenOnline, USA) and has been supported by the Austrian Science Funds (FWF). B. Bohle has been supported by the Austrian Science Funds and by the Christian Doppler Laboratory for Immunomodulation. M. Wallner has been supported by the Priority Program of the University of Salzburg and the Austrian National Bank (ÖNB). F. Stolz and A. Neubauer are employees of Biomay AG. The remaining authors declare that they have no relevant conflicts of interest.

## AUTHOR CONTRIBUTIONS

F. Ferreira, M. Wallner involved in conception and design of the study and supervision of the project. M. Wolf, L. Aglas, P. Briza, I. Aloisi, S. Huber, H. Hofer, M. Steiner, Maria A. Parigiani, Manuel Reithofer, and Beatrice Jahn‐Schmid acquired the data. M. Wolf, F. Ferreira, M. Wallner, P. Briza, and Beatrice Jahn‐Schmid analyzed and interpreted the data. T. E. Twaroch, A. Neubauer, F. Stolz, C. Ebner, B. Bohle, M. Hauser, and Claudia Asam made contribution with reagents/materials/analysis tools. F. Ferreira, M. Wallner, M. Wolf, and Beatrice Jahn‐Schmid drafted the manuscript. All authors discussed the results and their implications, read and commented on the manuscript at all stages, according to their contributions.
