# Supplementary material for: Amb a 1 isoforms: Unequal siblings with distinct immunological features
Source: Allergy. 2017 Jun 14;72(12):1874–82. doi: 10.1111/all.13196 (PMC5700413; doi:10.1111/all.13196)
Supplement: Supplementary file 10 [file ALL-72-1874-s010.docx]

ONLINE SUPPORTING INFORMATION

**Amb a 1 isoforms: unequal siblings with distinct immunological features**

Martin Wolf ^1^, Teresa E. Twaroch ^2^, Sara Huber ^1^, Manuel Reithofer ^6^, Markus Steiner ^1,3^, Lorenz Aglas ^1^, Michael Hauser ^1^, Iris Aloisi ^4^, Claudia Asam ^1^, Heidi Hofer ^1^, Maria A. Parigiani ^1^, Christof Ebner ^5^, Barbara Bohle ^6^, Peter Briza ^1^, Angela Neubauer ^2^, Frank Stolz ^2^, Beatrice Jahn-Schmid ^6^, Michael Wallner ^1^, Fatima Ferreira ^1^

1) Department of Molecular Biology, University of Salzburg, Salzburg, Austria

2) Biomay AG, Vienna Competence Center, Vienna, Austria

3) Laboratory for Immunological and Molecular Center Research, Paracelsus Medical University, Salzburg, Austria

4) Department of Biological, Geological, and Environmental Sciences, University of Bologna, Bologna, Italy

5) Allergy Clinic Reumannplatz, Vienna, Austria

6) Department of Pathophysiology and Allergy Research, Medical University of Vienna, Vienna, Austria

Corresponding Author:

Fatima Ferreira, PhD

Department of Molecular Biology, University of Salzburg

Hellbrunnerstr. 34, A-5020 Salzburg, Austria

Tel: +43-662-8044-5016, Fax: +43-662-8044-745016

Email: Fatima.ferreira@sbg.ac.at

**Material and Methods**

**Purification of Amb a 1 isoforms**

6 g of *Ambrosia artemisiifolia* pollen (batch: 020511204 purchased from Allergon Healthcare, Ängelholm, Sweden) were extracted with 180 ml of 5 mM sodium phosphate (NaP) buffer pH 6.8, for 16h at 4°C. Crude pollen extracts were clarified by centrifugation at 4,000 x g for 20 min. In order to remove pollen phenolic compounds, the supernatant was treated twice with 8 g of extraction buffer-soaked polyvinylpyrrolidone (Sigma-Aldrich, St. Louis, USA), followed by a 5 min-centrifugation step (1,000 x g) after each addition. After filtering through a 0.45 µm syringe filter (GE Healthcare, Chicago, IL, USA), the extract was loaded onto a 15 ml column packed with CHT Type I resin (Bio-Rad Laboratories Inc., Hercules, CA, USA) and eluted with a 4-segment linear gradient (0 to 500 mM NaP buffer pH 6.8 final concentration). Fractions containing Amb a 1.02 were pooled and dialyzed against 10 mM NaP pH 8.5. The flow through as well as early fractions containing a mix of Amb a 1.01 and Amb a 1.03 were adjusted to pH 9.5 with 1 M Tris buffer and loaded onto a 5 ml QHP Sepharose column (GE Healthcare) equilibrated with 50 mM Tris pH 9.5 and eluted with a 3 segment linear gradient (0-20% in 200 ml; 20-50% in 100 ml; 50-100% in 50 ml) from 0 to 1M NaCl in 50mM Tris pH 9.5. Fractions containing either Amb a 1.01 or Amb a 1.03 were pooled and dialyzed against 10mM NaP pH 8.5. Purified proteins were stored at -20°C. In the manuscript, nAmb a 1.01 is referred to as Amb a 1.01, nAmb a 1.02 as Amb a 1.02, and rAmb a 1.03 as Amb a 1.03.

Recombinant Amb a 1.03 was supplied by Biomay AG (Vienna, Austria). In brief, the protein was produced by heterologous expression in *P. pastoris*, purified by using a two-step chromatographic procedure and lyophilized in 5 mM NaP buffer, pH 7.4.

Prior to animal immunizations, protein samples were tested for their endotoxin content using the HEK-Blue^TM^ mTLR4 reporter cell assay (Invivogen, San Diego, CA, USA), according to manufactures instructions.

**Protein purification (Amb a 8 and Amb a 9)**

Recombinant Amb a 8 was expressed from a pET26b (Novagen, Merck KGaA, Darmstadt, Germany) construct in *Escherichia coli* BL21 StarTM (DE3) cells (Invitrogen, Carlsbad, CA, USA). Cells were grown at 37°C in LB supp. medium (1% (w/v) peptone, 0.5% (w/v) yeast extract, 0.5% (w/v) NaCl, 2 mM MgSO4, 1% (v/v) glycerol, 0.2% (w/v) ammonium sulfate, 10 mM sodium phosphate pH 7.4) supplemented with 25 mg/L kanamycin to an OD_600_ of 0.8, induced with 0.5 mM isopropyl-b-D-thiogalactopyranoside and expression was continued for 18h at 16°C. Cells were harvested by centrifugation and cell pellets were dissolved in 1/50 culture volume PBS pH 7.4 and cell breakage was performed using liquid nitrogen. After centrifugation at 15,000 x g, 10 mM EDTA and 5mM DTT were added under stirring to the supernatant. The extract was filtered through a 0.45 μm filter, applied to a poly-L-proline column, and eluted with PBS pH 7.4, 6 M urea. Final purification was performed by size exclusion chromatography in 10 mM sodium phosphate buffer pH 8 using a Superdex 75 10/300 GL column (GE Healthcare Biosciences). Recombinant proteins were lyophilized and stored at -20°C.

Amb a 9 inserted into the vector pET28b (EMD Biosciences Inc, San Diego, CA, USA) was transformed in *Escherichia coli* BL21 StarTM (DE3) cells (Invitrogen, Carlsbad, CA, USA) and grown in ZYM 5052 auto-inducing medium (1) (1%(w/v) N-Z-amine AS, 0.5% (w/v) yeast extract, 25 mM Na_2_HPO_4_, 25 mM KH_2_PO_4_, 50mM NH_4_Cl, 5 mM Na_2_SO_4_, 2 mM MgSO_4_, 0.5% (v/v) glycerol, 0.05% (w/v) glucose, 0.2% (w/v) lactose, 1 x trace metals) supplemented with 25 mg/L kanamycin for 18h at 37°C. Harvested cells were dissolved in 1/50 culture volume 25 mM tris/HCl pH 8.5 and cell breakage was performed using liquid nitrogen. After centrifugation at 15,000 x g, the solution was filtered through a 0.45 μm filter and protein purification was performed by anion-exchange chromatography using a 5 mL Q-sepharose column (GE Healthcare Biosciences, Little Chalfont, UK). The protein was eluted with 25 mM tris/HCl pH 8.5, 4% (v/v) 2-propanol, 500mM NaCl. Final purification was performed by size exclusion chromatography in 10 mM sodium phosphate buffer pH 8 using a Superdex 75 10/300 GL column (GE Healthcare Biosciences). Recombinant proteins were lyophilized and stored at -20°C.

**Physicochemical characterization**

Amino acid composition of the protein preparations was analyzed by amino acid analyses according to the PicoTag^TM^ method (Waters, Milford, MA, USA) using a HP1100 HPLC system (Hewlett-Packard, San Jose, CA, USA) equipped with a 3.9 x 150mm Novapak C_18_ column (Waters). Protein dispersity and aggregation behavior was assayed by dynamic light scattering (DLS) using a DLS 802 system (Viscotek Corp., Houston, TX, US). Data of 10 measurements were accumulated for evaluation with the OmniSize™ software and displayed as mass weighted distribution of the hydrodynamic radius. Circular dichroism (CD) spectra were recorded with a JASCO-J815 spectropolarimeter (Jasco, Tokyo, Japan) fitted with a PTC-423S Peltier type single position cell holder in 10 mM NaP buffer pH 8 at 20°C or 95°C, respectively. Samples were measured at 0.1 mg/ml from 190-260nm at resolution of 1 nm with 1 nm bandwidth and a scanning speed of 1 nm/s. Five spectra were averaged and background corrected. Data are presented as mean residue molar ellipticity. Infrared spectra of the protein preparations were recorded at a constant temperature (25°C) using an AquaSpec transmission cell adapted to a Tensor II Confocheck Fourier transformed infrared (FTIR) system (Bruker Optics Inc., Billerica, MA, USA). Second derivatives were calculated by applying the Savitzky–Golay algorithm with 25 smoothing points. Spectra were vector-normalized and baseline corrected. For the analysis of secondary structure elements, the Quant2 method provided by Confocheck (Bruker Optics Inc.) was used.

**Peptide analysis by nano-LC-MS/MS**

1 μg protein of pollen extracts and purified preparations were digested with the ProteoExtract All-in-One Trypsin Digestion Kit (EMD Millipore, Billerica, MA, USA). After the digest, samples were desalted using C_18_ ZipTips (EMD Millipore, Billerica, MA, USA). Resulting peptides were separated by reverse-phase nano-HPLC (Dionex Ultimate 3000, Thermo Fisher Scientific, Bremen, Germany, column: PepSwift Monolithic Nano Column, 100 μm x 25 cm, Dionex). The column was developed with an acetonitrile gradient (Solvent A: 0.1% (v/v) FA/0.01% (v/v) TFA/5% (v/v) ACN; solvent B: 0.1% (v/v) FA/0.01% (v/v) TFA/90% (v/v) ACN; 5–45% B in 60 min) at a flow rate of 1 μl/min at 55°C). The HPLC was directly coupled via nano electrospray to a Q Exactive Orbitrap mass spectrometer (Thermo Fisher Scientific). Capillary voltage was 2 kV. For peptide identification, a top 12 method was used with the normalized fragmentation energy at 27%. Survey and fragment spectra were analyzed with Proteome Discoverer version 1.4 with Sequest as search engine (Thermo Fisher Scientific) or Peaks Studio 8 (Bioinformatics Solutions, Waterloo, Canada), respectively. Databases used for general searches (e.g. search for contaminants) were UniProt (SwissProt/TrEMBL) and NCBI (release dates July 2016). For specific searches, an in-house database consisting of all *Ambrosia artemisiifolia* allergens compiled from www.allergen.org was used. In ProteomeDiscoverer, search parameters were: FDR 1%, only peptides with XCorr <2.3 and confidence higher than ‘Medium’ were considered. In PEAKS, an FDR of 1% was used, as well. Since FDR filtering with small databases is not applicable, peptides for specific searches were selected based exclusively on their -10lgP score. Only peptides with a score ≥35 were used. The parameters for both programs provide search results with very high confidence. For a semi-quantitative determination of the purity and isoform content of samples, the precursor ion intensity of diagnostic peptides as determined by Proteome Discoverer was used.

**Immunoblots, ELISA, and Inhibition ELISA**

Proteins were separated by 1 or 2D SDS-PAGE using 15% polyacrylamide gels. 2D gels were performed with a pH gradient from 4-7 using the ReadyStrip™ IPG Strip system from Biorad (Biorad, Hercules, CA, USA). Thereafter, proteins were either detected by Coomassie Brilliant Blue G-250 (Biorad, Hercules, CA, USA) or blotted onto a nitrocellulose membrane (Whatman, Maidstone, UK). Blots were incubated with a 1:10 diluted serum pool of 10 ragweed allergic patients and bound IgE was detected using an AP-conjugated anti-human IgE antibody (1:10000) (BD Biosciences, Franklin Lakes, USA). For ELISA experiments, Maxisorp plates (Nunc, Thermo Fisher, Waltham, MA, USA) were coated with 100 ng purified protein /well or 500 ng pollen extract/well in 50µl PBS pH 7.4 overnight at 4°C. After washing and blocking, plates were either incubated with diluted human sera (1:50 for IgG and 1:250 for IgE detection) (buffer: TBS, pH 7.4, 0.05% (v/v) Tween, 1% (w/v) BSA) or with serial dilutions of mouse sera (buffer: TBS, pH 7.4, 0.05% (v/v) Tween, 1% (w/v) BSA) overnight at 4°C. Bound human IgE was detected using an AP-conjugated mouse anti-human IgE antibody (Clone G7-26) (BD Biosciences, Franklin Lakes, USA), human IgG1 was detected by an AP-conjugated mouse anti-human IgG1-hinge antibody (Clone 4E6) and human IgG4 was detected using an AP-conjugated mouse anti-human IgG4 pFc’ antibody (Clone HP6023). Mouse IgG1 was detected by an AP-conjugated rat anti-mouse IgG1 antibody (Clone SB77e) and mouse IgG2a was detected using an AP-conjugated rat anti-mouse IgG2a antibody (Clone SB84a) (all Southern Biotech, Birmingham, USA). After incubation for 1h at 37°C and 1h at 4°C, colorimetric detection with 10 mM 4-nitrophenyl phosphate (Sigma-Aldrich, St. Louis, MO, USA) was performed. All measurements were done in duplicates and results presented as mean OD values.

# For inhibition-ELISA experiments, sera were diluted (1:100 for cross-inhibition and 1:200 for extract inhibitions) and pre-incubated overnight with inhibitor protein at concentrations of 50µg/ml to 50ng/ml in 1:10 dilution-steps for cross-inhibition between natural and recombinant versions of Amb a 1.03. For cross-inhibitions among all three isoforms, concentrations of 1µg/ml to 100pg/ml in 1:10 dilution-steps were employed. For inhibition of coated extracts, a fixed inhibitor concentration of 10µg/ml was used. Mouse anti human IgE-HRP (Clone B3102E8) (Southern Biotech, Birmingham, USA) and BM Chemiluminescence ELISA Substrate (POD) (Roche AG, Basel, Switzerland) were used for detection of bound antibodies.

**Mediator release assays**

For mediator release assays, RBL-2H3 cells transfected with the alpha chain of the human IgE receptor were passively sensitized with IgE antibodies from ragweed allergic donors (human IgE analyses) (2). Alternatively, for the analysis of mouse IgE antibodies, non-transfected RBL-2H3 (ATCC® CRL2256™) were used and passively sensitized with mouse blood samples. After several washing steps, mediator release was triggered by addition of diluted protein antigens (1 ng/ml - 100 µg/ml). Antigen-dependent β-hexosaminidase release into the supernatant was measured by enzymatic cleavage of the fluorogenic substrate 4-methylumbelliferyl-N-acetyl-β-D-glucosaminide and expressed as % of total enzyme content of Triton X100-treated cells.

**Facilitated antigen binding assay (FAB)**

FAB assays were performed according to Shamji *et al*. and Francis *et al*. (3, 4). An indicator serum pool, obtained by mixing equal amounts of sera from four Amb a 1-allergic patients, was used in the experiments. 20 µl indicator serum were mixed with 5 µl (c= 0,1 µg/ml) antigen solution and 15 µl of a test serum. The mix was incubated for 1h at 37°C and thereafter 1x10^5^ EBV transformed B-cells were added for 1h at 4°C. Cells were stained with a 1:10 dilution of goat anti-human IgE-FITC antibody (KPL, Gaithersburg, USA) in PBS pH 7.4, 0.5% BSA, for 30 min at 4°C. Anti-CD23 was detected using a 1:20 dilution of a mouse anti-human CD23-PE antibody (BD Pharmingen, Becton, Dickinson and Company Becton Drive Franklin Lakes, NJ, USA). Flow cytometry measurements were performed on a FACS Canto II (Becton, Dickinson and Company).

**Proliferation assays**

PBMC from ragweed-allergic individuals had been isolated in a prior study from peripheral blood during or shortly after the ragweed pollen season and cryopreserved (5). Here, PBMC were thawed, washed and stimulated for 6 days with Amb a 1.01, Amb a 1.02 or rAmb a 1.03 at equivalent concentrations. IL-2 at 20U/ml was used as positive control. At day 6, proliferation was assessed by the addition of ^3^H-thymidine for 16 hours. Then cells were harvested and ^3^H-thymidine uptake was measured by scintillation counting. Stimulation indices (SI) were calculated as the ratio of cpm (counts per minute) of stimulated cultures to cpm of unstimulated cultures. Background counts (medium alone) ranged between 1,555 and 18,470 cpm.

**Amb a 1-specific T cell clones**

T cell clones (TCC) had been established as previously described (5) and cryopreserved. Briefly, ragweed extract was used to stimulate PBMC and after 3 days 10U/ml IL-2 were added to expand activated T cells. At day 9, T cell blasts were enriched by Ficoll gradient centrifugation, further expanded by addition of IL-2 and further splitted by feeder plus IL-2. TCC were obtained by limiting dilution, screened for Amb a 1.03-reactivity and epitopes recognized were determined by stimulation with 12mer synthetic peptides covering the amino acid sequence of Amb a 1.03 overlapping by 9aa. Five TCC were thawed, shortly expanded by feeder plus IL-2. After resting for 7 days TCC were stimulated by the 3 Amb a 1 isoforms at 0.5ug/ml, the relevant peptides or 40 U/IL-2 as control for 2 days and proliferation was measured by ^3^H-thymidine uptake. Stimulation indices (SI) were calculated as described above.

**References**

1. Studier FW. Protein production by auto-induction in high density shaking cultures. *Protein Expr Purif* 2005;**41**(1):207-234.

2. Vogel L, Luttkopf D, Hatahet L, Haustein D, Vieths S. Development of a functional in vitro assay as a novel tool for the standardization of allergen extracts in the human system. *Allergy* 2005;**60**(8):1021-1028.

3. Francis JN. The facilitated antigen binding (FAB) assay--a protocol to measure allergen-specific inhibitory antibody activity. *Methods Mol Med* 2008;**138**:255-261.

4. Shamji MH, Wilcock LK, Wachholz PA, Dearman RJ, Kimber I, Wurtzen PA, et al. The IgE-facilitated allergen binding (FAB) assay: validation of a novel flow-cytometric based method for the detection of inhibitory antibody responses. *J Immunol Methods* 2006;**317**(1-2):71-79.

5. Jahn-Schmid B, Hauser M, Wopfner N, Briza P, Berger UE, Asero R, et al. Humoral and cellular cross-reactivity between Amb a 1, the major ragweed pollen allergen, and its mugwort homolog Art v 6. *J Immunol* 2012;**188**(3):1559-1567.

**Supplementary table legends**

**Table S1**

List of patients´ sera used in the present study. n.d., not detected.

**Table S2.** Mass spectrometry analyses of tryptic digests of ragweed pollen extract. Relative quantification of Amb a 1 isoforms was performed by analysis of the total precursor ion current of all isoform-specific peptides.

**Table S3.** Mean antibody titers of immunized animals.

**Supplementary figure legends**

**Figure S1.** Sequence alignment (A) and sequence identities of Amb a 1 isoforms (B). Amb a 1.01 refers to Amb a 1.0101, Amb a 1.02 to Amb a 1.0201, Amb a 1.03 to Amb a 1.0301, Amb a 1.04 to Amb a 1.0401, and Amb a 1.05 to Amb a 1.0501, respectively. Signal peptide sequences are shaded in gray. Protein surface charges (C). Three-dimensional models of Amb a 1 isoforms based on the structure of Jun a 1 (PDB: 1PXZ) allergen from *Juniperus ashei*. Surfaces are colored according to calculated charges. Blue, negatively charged surfaces; Red, positively charged surfaces.

**Figure S2.** SDS-PAGE and Coomassie staining of purified Amb a 1 preparations and ragweed pollen extract; 1.01, natural Amb a 1.01; 1.02, natural Amb a 1.02; r1.03, recombinant Amb a 1.03; n1.03, natural Amb a 1.03 (A). The isoform composition of the Amb a 1 preparations was analyzed by MS (B).

**Figure S3.** Mediator release of RBL cells passively sensitized with serum IgE from Amb a 1-allergic patients stimulated with increasing concentrations of Amb a 1 isoforms. Titration curves for two representative patients are shown (A). Cross-inhibition ELISA was performed using sera from Amb a 1-allergic patients (n= 3). Titration curves for the different isoforms are shown in the left graph using serum from one allergic donor (patient 22) (B). IgE immunoblot of purified natural (n) and recombinant (r) Amb a 1.03 isoform using sera from five allergic donors (C).

**Figure S4.** CD spectra of Amb a 1 isoforms were recorded at 20 and 95°C (A). FTIR measurements were performed at 25°C (B). FTIR data were evaluated using the second derivative of the amide I band (C). Secondary structure content was calculated using the CONFOCHECK software bundle (D).

**Figure S5.** Patients´ (n= 39) antibody-binding to ragweed pollen extracts was analyzed by immunoblot (A) and ELISA (B). +AIT, ragweed-allergic patients subjected to allergen-specific immunotherapy; -AIT, non-treated ragweed-allergic patients; n.A, non-allergic donor.

**Figure S6.** Cross-inhibition capacity was assessed in ELISA by pre-incubating sera from ragweed-allergic patients with increasing concentrations of the different Amb a 1 isoforms. Percentage of inhibition is given taking the values obtained with serum without added inhibitor as reference. Titration curves for three representative patients are shown.
